# Brain fogginess, gas and bloating: a link between SIBO, probiotics and metabolic acidosis

**DOI:** 10.1038/s41424-018-0030-7

**Published:** 2018-06-19

**Authors:** Satish S. C. Rao, Abdul Rehman, Siegfried Yu, Nicole Martinez de Andino

**Affiliations:** Division of Gastroenterology/Hepatology, Department of Internal Medicine, Medical College of Georgia, Augusta University, Augusta, GA USA

## Abstract

**Background:**

D-lactic acidosis is characterized by brain fogginess (BF) and elevated D-lactate and occurs in short bowel syndrome. Whether it occurs in patients with an intact gut and unexplained gas and bloating is unknown. We aimed to determine if BF, gas and bloating is associated with D-lactic acidosis and small intestinal bacterial overgrowth (SIBO).

**Methods:**

Patients with gas, bloating, BF, intact gut, and negative endoscopic and radiological tests, and those without BF were evaluated. SIBO was assessed with glucose breath test (GBT) and duodenal aspiration/culture. Metabolic assessments included urinary D-lactic acid and rblood L-lactic acid, and ammonia levels. Bowel symptoms, and gastrointestinal transit were assessed.

**Results:**

Thirty patients with BF and 8 without BF were evaluated. Abdominal bloating, pain, distension and gas were the most severe symptoms and their prevalence was similar between groups. In BF group, all consumed probiotics. SIBO was more prevalent in BF than non-BF group (68 vs. 28%, *p* = 0.05). D-lactic acidosis was more prevalent in BF compared to non-BF group (77 vs. 25%, *p* = 0.006). BF was reproduced in 20/30 (66%) patients. Gastrointestinal transit was slow in 10/30 (33%) patients with BF and 2/8 (25%) without. Other metabolic tests were unremarkable. After discontinuation of probiotics and a course of antibiotics, BF resolved and gastrointestinal symptoms improved significantly (*p* = 0.005) in 23/30 (77%).

**Conclusions:**

We describe a syndrome of BF, gas and bloating, possibly related to probiotic use, SIBO, and D-lactic acidosis in a cohort without short bowel. Patients with BF exhibited higher prevalence of SIBO and D-lactic acidosis. Symptoms improved with antibiotics and stopping probiotics. Clinicians should recognize and treat this condition.

## Introduction

Abdominal bloating, gas and distension are common gastrointestinal symptoms that are caused by many conditions including carbohydrate intolerance and small intestinal bacterial overgrowth (SIBO)^1^. Brain Fogginess (BF) describes a constellation of symptoms comprised of mental confusion, impaired judgment, poor short-term memory, and difficulty with concentration, which is often transient and disabling. Previously, similar symptoms, along with slurred speech and gait disturbances have been described in patients with short bowel syndrome^2,3^. These patients were found to have metabolic acidosis with elevated levels of D-lactic acid in the serum. Others have described brain fogginess in association with other chronic disorders including postural orthostatic tachycardia syndrome^4,5^. Recently, probiotic use has been implicated in the production of D-lactic acidosis, both in short bowel syndrome patients and in the first 2 weeks of life in infants who were fed probiotic-containing formula^6,7^. Typically, D-lactic acidosis is caused by the fermentation of ingested carbohydrate by D-lactic producing bacteria such as lactobacillus and bifidobacterium in the bowel^2,3^.

In a preliminary report, we described seven patients who presented with both unexplained abdominal bloating and BF and who were consuming probiotics^8^. Whether there is a link between abdominal bloating, distention, gas, D-lactic acidosis, and small intestinal bacterial overgrowth (SIBO), and probiotic usage is not known. We examined whether small intestinal bacterial overgrowth (SIBO), particularly with D-lactic acid producing bacteria may cause a syndrome of BF, gas, bloating, and neurocognitive symptoms.

Our aim was to evaluate a consecutive series of patients with unexplained BF, abdominal bloating and gas, for SIBO, using both duodenal aspirate and cultures, and a glucose breath test (GBT), and simultaneously assess for metabolic acidosis.

## Methods

We conducted a prospective observational study of consecutive adult patients referred to our tertiary care center over 3 years. Patients presenting with unexplained abdominal gas, bloating and distension, and BF were included. BF was defined as presence of two or more of the following symptoms for more than 3 months during their initial clinic visit: mental confusion, cloudiness, impaired judgment, poor short-term memory, and difficulty with concentration. Given the multiple constellation of abdominal symptoms, our patients could not be definitively categorized into any one of the Rome III classification of functional gastrointestinal disorders, and hence they were categorized as unexplained symptoms. Additionally only subjects with normal upper endoscopy and duodenal biopsy, normal colonoscopy and biopsy, and normal abdominal CT imaging, and normal hematological and biochemical profiles were enrolled. Patients with a history of small bowel or colonic surgery, those with known intestinal dysmotility (scleroderma or pseudo-obstruction), and those with a history of recent antibiotic use (in the previous 6 weeks), or history of known neurological or neuropsychiatric problems or celiac disease or Helicobacter Pylori infection or renal or liver failure were excluded from the study. We also evaluated a cohort of patients with similar symptom profiles and who met the inclusion and exclusion criteria, but without brain fogginess (Non-BF). The institutional review board approved this study, number 611925–2.

All patients filled out a validated questionnaire^9^ that assessed 9 symptoms; abdominal pain, belching, bloating, fullness, indigestion, nausea, diarrhea, vomiting and gas. The frequency, intensity and duration of each symptom was rated on a 0–3 point Likert scale^8–10^. A mean total score was calculated, and symptoms were assessed at baseline and after treatment. A score ≥5 was considered severe. All patients were followed up for 6 months. Additionally, we inquired about probiotic use, a history of food fads, yogurt consumption, and use of over the counter medications and recent antibiotic use. Patients were asked to discontinue the use of laxatives and drugs that affect intestinal motility (opioids, anticholinergics, and anti-diarrheals) at least one week prior to the study, but were allowed to continue with other maintenance medications.

Patients had duodenal aspirate and cultures and glucose breath test within one week^10–12^. No new medications were allowed during testing. Blood samples were collected for bun, electrolytes, creatinine and liver biochemistry. Metabolic testing included assessment of blood glucose, insulin, ammonia and L-lactate levels and urinary D-lactic acid levels after a carbohydrate challenge. Assessment of gastrointestinal transit consisted of the wireless motility capsule test (SmartPill ®, Medtronic Corporation, Minneapolis, MN) where a subject ingested a capsule containing pressure, pH, and temperature sensors, and wore a recorder for 5 days to assess regional and whole gut transit^13,14^. Alternatively, they had a gastric emptying test with radioactive technetium meal and a colonic transit test where the subject ingested a capsule with 24 radio-opaque markers and had a plain abdomen X-ray at 120 h^13,14^.

### Duodenal aspiration and culture

After an overnight fast, an upper endoscope was passed into the distal duodenum with minimal air insufflation. Using aseptic technique, a 2 mm nasobiliary catheter (Liguory ®, COOK Medical, Bloomington, IN, US) was advanced through the endoscope into the distal duodenum, and 3–5 ml of fluid was aspirated and sent to the microbiology laboratory for standard aerobic, anaerobic and fungal cultures. The duodenal culture results were considered as positive for SIBO if one or more organisms (aerobic or anaerobic) were cultured with a colony count of ≥10^3^ CFU/ml^10,11^.

### Glucose breath test (GBT)

After an overnight fast, 75 g of glucose dissolved in 250 ml water was administered orally. Breath samples were obtained at baseline and at 15 min intervals for 2 h, and analyzed for both hydrogen and methane using gas chromatography (QuinTron Micro Analyzer ®, QuinTron Instrument Company Inc. Milwaukee, WI)^10–12^. Throughout the study, the patients scored the presence and severity of symptoms on a likert scale^7^. GBT was considered positive for SIBO if there was ≥20 p.p.m. increase above baseline for H_2_ or for combined H_2_ and CH_4_^11,15–18^. In 3 subjects with diabetes, fructose breath test was performed using previously described methods^19^.

### Metabolic testing (lactic acid)

In order to assess lactic acidosis we used a novel method of assessing blood and urine lactic acid levels after an oral carbohydrate challenge (Glucose) i.e., simultaneously alongside the glucose breath test. Blood samples were collected at baseline, 1/2, 1, 2, and 3 h after administration of glucose, and sent for serum L-lactic acid, and ammonia levels. L-Lactic acid levels ≥2.2 mmo/L were considered positive and indicative of acidosis. Urine samples for D-lactic acid were collected at baseline, 1 h, and 3 h during and after the GBT and analyzed in specialist lab (Mayo Clinic Laboratories, Rochester, MN). D-Lactic acid levels ≥0.22 mmol/L were considered abnormal and indicative of acidosis. Urinary as opposed to blood D-lactic acid assessment was chosen due to its stability at room temperature and for remote processing as recommended by the specialist lab.

### Statistical analysis

Symptoms and global satisfaction data were compared before and after treatment using Chesapeake Test. CHI square test was used to compare data between the BF and non-BF groups with regards to the prevalence of D-lactic acidosis, and SIBO and symptoms.

## Results

### Demographics

**Fig. 1:**
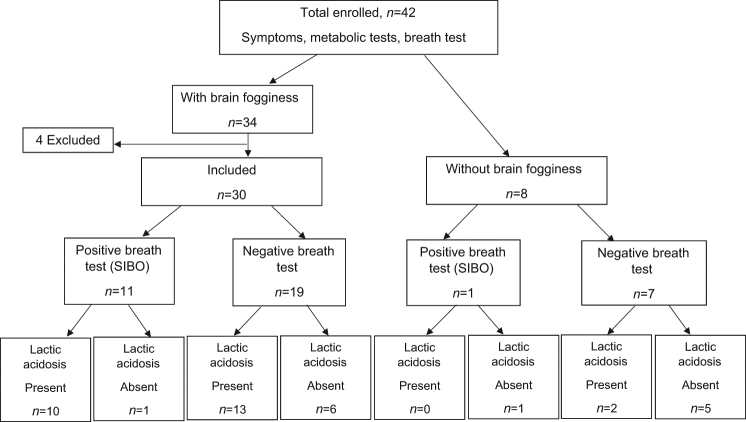
Consort flow diagram describing enrollment and disposition of metabolic and breath test results. *SIBO* small intestinal bacterial overgrowth

We evaluated a total of 42 patients, of whom 34 had BF and 8 had no BF (non-BF) (Fig. 1). Four patients in the BF group were excluded because of recent antibiotic use (1), inability to complete all tests or technical problems with collection/transport of samples (2), and new diagnosis of Parkinson’s disease (1). Thirty patients, f/m = 19/11, mean age 52 years were included (Table 1A). Six patients were on gluten-restricted, 3 on FODMAP-restricted, and 1 on Paleo diets. All 8 patients (f/m = 5/3, mean age 49 years) in the non-BF group met inclusion criteria (Table 1B). Two patients were on FODMAP restricted diet.

**Table 1:** A: This shows key demographic features, medication use, occurence of brain fogginess during breath test, D and L-lactic acid levels, and results of duodenal culture and glucose breath test in the brain fogginess group

Patient	Age (Yrs)	Gender	PPI Use	Opiate Use	Brain Fog Reproduced on breath test	Baseline Urinary D-lactic acid (mmol/L)	Peak Urinary D-lactic acid level (mmol/L) NR = < 0.22	Peak Serum L-lactic acid level (mmol/L) NR = < 2.2	Duodenal Culture results	Glucose Breath test
1	41	Female	No	No	Yes	0	0.24	5.1	Negative	Negative
2	47	Female	No	No	Yes	0.1	0.1	0.6	SIBO	Negative
3	52	Female	No	Yes	Yes	0.3	0.57	2.8	NA	Negative
4	49	Female	No	No	Yes	0.1	0.31	1.6	SIBO	Negative
5	74	Male	Yes	Yes	Yes	0	0.13	1.8	SIBO	Negative
6	45	Female	No	No	Yes	0	0.12	1.3	SIBO	Negative
7	40	Female	No	No	Yes	0.1	0.38	1.3	Negative	Negative
8	55	Male	No	No	No	0.1	0.28	2.9	SIBO	Positive
9	52	Male	No	No	Yes	0	0.24	2.2	Negative	Negative
10	50	Female	Yes	Yes	Yes	0	0.1	1	SIBO/SIFO	Positive
11	47	Female	No	No	No	0	0.22	1.6	SIBO/SIFO	Positive
12	37	Male	Yes	Yes	Yes	0.1	0.24	1.6	Negative	Negative
13	50	Female	Yes	No	No	0	0.26	2.4	SIBO/SIFO	Positive
14	80	Male	No	Yes	No	0	0.13	1.2	Negative	Negative
15	38	Female	No	No	Yes	0	0.48	NA	Negative	Positive
16	63	Female	Yes	No	Yes	0.1	0.23	1.2	Negative	Negative
17	69	Female	No	No	Yes	0	0.57	1.4	SIBO	Positive
18	81	Female	Yes	No	Yes	0.14	0.22	2.4	Negative	Negative
19	81	Male	Yes	No	No	0.14	0.34	1.7	Negative	Positive
20	41	Male	Yes	No	No	0.12	0.19	1.8	SIBO	Negative
21	20	Male	No	No	Yes	0	0.23	1.6	Negative	Negative
22	22	Female	No	Yes	No	0.1	0.24	1.3	Negative	Negative
23	61	Female	Yes	No	Yes	0.13	0.23	1	SIBO	Negative
24	54	Male	Yes	Yes	No	0.15	0.25	1.6	Negative	Negative
25	32	Female	Yes	No	No	0.14	0.47	1.9	Negative	Positive
26	44	Male	Yes	No	Yes	0.16	0.24	2.7	Negative	Positive
27	66	Female	No	No	Yes	0.19	0.33	3.1	SIBO	NA
28	63	Female	No	No	No	0	0.22	0.9	SIBO	Positive
29	26	Male	No	No	Yes	0.11	0.15	1.8	SIBO	Negative
30	66	Female	Yes	No	Yes	0.1	0.33	2.3	NA	Positive

B: Demographics and results in the Non-brain fogginess group										
1	61	Male	No	no	No	0.14	0.20	0.8	Negative	Negative
2	44	Female	Yes	Yes	No	0.3	0.6	2.0	SIBO	Negative
3	53	Male	no	no	No	0.20	0.32	1.2	Negative	Negative
4	44	Female	no	no	No	0.18	0.34	2.3	Negative	Negative
5	59	Female	Yes	no	No	0.1	0.1	0.8	SIBO/SIFO	Positive
6	27	Male	no	no	No	0.12	0.19	0.9	Negative	Negative
7	21	Female	no	no	No	0.18	0.19	0.9	Negative	Negative
8	48	Female	Yes	no	No	0.15	0.16	1.4	Negative	Negative

NA technical failure

### Gastrointestinal symptoms

The most common symptoms in the BF group were abdominal pain (96.7%), bloating (93.3%), distention (90%), fullness (90%), gas (90%), cramping (80%), diarrhea (76.7%), and belching (90). Severe bloating with significant and rapid increase in abdominal size from normal to full distension within few minutes was described by 6/30 (20%) patients in BF group only. In the non-BF group, the most common symptoms were abdominal pain (75%), bloating (100%), distention (88%), fullness (88%), gas (100%), belching (88%), diarrhea (50%), and cramping (75%). Severe symptoms (≥5), were pain, bloating, distention, and gas in both groups.

### Brain fogginess

Neurocognitive symptoms including BF or mental confusion or impaired judgment, poor short-term memory, and difficulty with concentration were described by all of the patients in the BF group. These symptoms lasted between 30 min to several hours, often post-prandial, and intermittently during the course of the day. Also 28/30 (94%) patients reported post-prandial fatigue and weakness, and 2/30 reported persistent daily fatigue unrelated to meal intake. BF was so severe that 4/30 (13.3%) had quit their jobs. One patient had recurrent near syncopal episodes after carbohydrate-rich meals. One patient had symptoms of bloating and BF with a blistering rash on the palms. Another patient’s symptoms caused her to be bed-ridden for up to a week after consuming a large carbohydrate-rich meal.

### Probiotics and other medication use

All patients in the BF group were taking probiotics (range 3 months to 3 years), some were taking 2–3 different varieties containing lactobacillus species, and/or bifidobacterium species or streptococcus *thermophillus* and others. Additionally 11 (36.7%) were using cultured yogurt daily, and 2 (6.7%) large amounts (20 oz.) of homemade cultured yogurt daily. Opioid use was found in 7/30 (23.3%), and PPI use and multivitamins in 13/30 (43.3%). Fish Oil and Biotin supplementation in 4/30 patients and 1/30 (3.3%) were taking ubiquinone, dessicated thyroid, simethicone, melatonin, curcumin, saw palmetto, samento extract, and artemisinin extract. One patient (12%) in the non-BF group took probiotics (Lactobacillus *rhamnosus*), 3/8 (37%) were using PPI, 3/8 (37%) multivitamin and fish oil supplements, and one opioids.

### Breath test

In the BF group, breath testing was positive for SIBO in 11/30 (36.6%) patients (Table 1, Fig. 1). The patient’s typical BF symptom(s) experienced at home was reproduced during the breath test in 20/30 (66%) and all of them had evidence of SIBO either with the breath test or with culture. In the non-BF group, breath test was positive in 1 (14%) and negative in 7 (16%); D-lactic acidosis was seen in 2/7 (28%) patients with negative breath test.

### Duodenal aspirate and culture

**Fig. 2:**
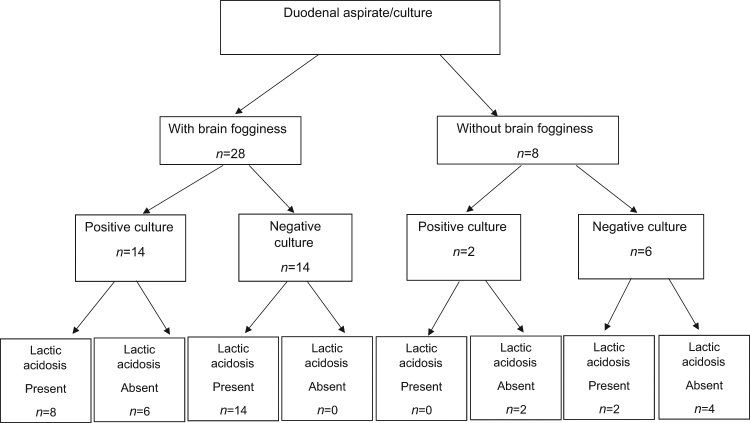
**Flow diagram describing the correlation between duodenal aspirate/culture results in both groups**

In the BF group, cultures for SIBO were positive in 14 (46.7%) patients and negative in 14 patients (46.7%) (Fig. 2); 2 patients did not have duodenal aspiration. Aerobic strains were grown in 22 (64.7%), and anaerobic bacteria in 9 (26.5%) patients. The predominant aerobic organisms were *Streptococcus species, Staphylococcus species, Neisseria species*, *and Hemophilus species*, and the predominant anaerobic organisms were gram negative rods, and cocci, gram positive rods – lactobacillus species, and prevotella species. The disposition of D and/or L-lactic acidosis is summarized in Fig. 2. In the non-BF group, 2/8 (25%) had positive culture for SIBO and one subject grew streptococcus species, and neisseria, and second subject grew rothia species and pseudomonas, and 1/8 (14%) grew candida species.

### Metabolic testing (lactic acid)

In the BF group, D-lactic acidosis was seen in 23 (76.7%) patients along with concurrent L-lactic acidosis in 9 (30%) patients (Table 1A). The mean BUN level was 13.5 mg/dl (normal <23), mean creatinine level was 0.74 mg/dl (normal <1.6), and mean ALT level was 27.8 U/l (normal <49). The mean baseline ammonia level was 23.4 umol/l (normal <35) with normal liver chemistries, but 9/30 (30%) had mild elevations (39–51 umol/l) of ammonia during the breath test. In the non-BF group, 2 (28%) patients had D-lactic acidosis and one of them also had l-lactic acidosis (Table 1B). The BUN and liver chemistry was normal and mean baseline ammonia level was 18.1 umol/l, and 2/8 (38–54 umol/l) had mild elevations during the breath test. The prevalence of D-lactic acidosis was significantly higher in the BF group when compared to the non-BF group (76.7 vs. 25%, *p* < 0.006). Also 10/13 (78%) PPI users and 4/7 opioid users had D-lactic acidosis.

### Gastrointestinal transit assessment

In the BF group, 17 patients had wireless motility capsule test (WMC), 13 had gastric emptying scintigraphy, and 7 had colonic transit study with radioopaque markers^12,13^. Overall 10/30 patients (33%) had abnormal gastrointestinal transit and evidence of dysmotiltiy. Gastroparesis was diagnosed in 5/30 (16.7%) patients, 1 of whom had concurrent prolonged small bowel transit time, and another concurrent slow colonic transit. Rapid gastric emptying was seen in 1 patient with normal small bowel or colonic transit, and the rest had normal gastric emptying. Slow transit constipation was found in 6/24 (25%) patients who had colonic transit studies, 1 of whom had gastroparesis, and another had slow small bowel transit. All 10 patients with dysmotility had evidence of SIBO and evidence for lactic acidosis. In the non-BF group (based on WMC), 2 patients (25%) had gastroparesis, and 3 patients (37%) had slow colonic transit; one of whom had concurrent upper dysmotility. There was no difference (*p* ≥ 0.2) in prevalence of abnormal transit between groups.

### Testing summary

In the BF group, metabolic testing for D-lactic acid/L-lactic acid production was positive in 24/30 (80%) patients, duodenal aspirate/culture was positive in 14 (46.7%), and glucose BT in 11 (36.6%). Based on either a positive GBT and/or duodenal aspirate/culture, 19 (63.3%) patients were diagnosed with SIBO and all of them had d-lactic acidosis. The prevalence of SIBO was significantly higher in the BF group when compared to the non-BF group (63.3 vs. 25%, *p* = 0.05).

### Follow up

**Fig. 3:**
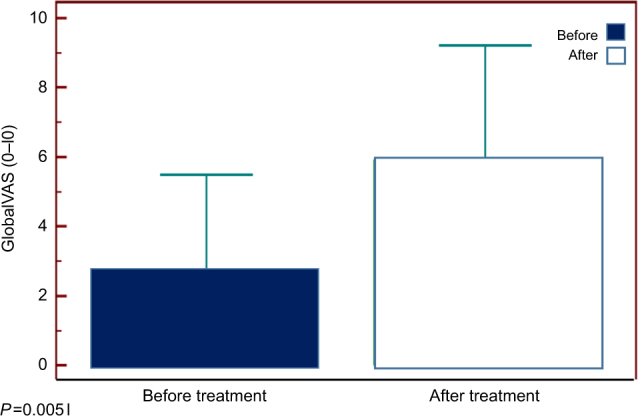
**Improvement in gastrointestinal symptoms after treatment as assessed by Global VAS Score in patients with brain fogginess**

In the BF group, all patients with evidence of lactic acidosis and/or SIBO were treated with various antibiotics (amoxicillin, amoxicillin-clavulanic acid, cotrimoxazole, rifaximin, metronidazole, tinidazole, and ciprofloxacin) based on the individual allergy profile and culture/sensitivity results, and were asked to discontinue probiotics (*N* = 23). The remaining patients without evidence of SIBO were asked to discontinue probiotics and yogurt use (*N* = 7). 24/30 (80%) attended follow-up clinic visits at 6 weeks and 3 months and rest were assessed by phone inquiry. GI symptoms were inquired through a validated questionnaire^9^, including global improvement using a 0–10 visual analog scale. We also inquired about the presence or absence of BF. After treatment, 70% of patients reported significant improvement in their symptoms with a significant change in the mean global VAS score (*P* = 0.005), (Fig. 3). 85% of patients reported complete resolution of BF. There were also significant (*p* < 0.05) improvements in the individual mean symptom scores (before vs. after) for abdominal pain (6.17 vs. 4.17), cramping (3.17 vs. 2.17), bloating (6.5 vs. 4.08), fullness (5.58 vs. 3.5), and distention (5.75 vs. 3.75); but not for other symptoms. Likewise, in the non-BF group, all 4 patients with SIBO/D-lactic acidosis received antibiotics and reported significant improvement in symptoms at 3-month follow-up; abdominal pain (7.2 vs 2.2), cramping (4.1 vs 2), bloating (7.8 vs 3.1), fullness (4.9 vs 2.4), and distension (6.1 vs 3.2). There was no difference in symptom(s) improvement between the two groups. (*p* > 0.2). Mean global satisfaction (VAS) score also improved (2.0 ± 1.3 vs. 7.4 ± 2.1, *p* < 0.05).

## Discussion

We describe a cohort of patients with an intact gut who reported brain fogginess associated with unexplained abdominal bloating, pain, gas and distension, and in whom there was evidence of probiotic use, D-lactic acidosis and SIBO. The prevalence of both D-lactic acidosis and SIBO was significantly higher in patients with BF compared to those without BF.

The unique feature of this cohort was the presence of BF, which was described as variable in severity and intensity, often post-prandial, and forced some patients to quit their jobs. Also they reported significant bloating, abdominal pain, distention, and fullness. Additional symptoms included extreme fatigue, restlessness, weakness, and disorientation. These symptoms occurred at variable times and could last 30 min to a few hours. Few described excessive belching, diarrhea, abdominal cramping and headache. During the breath test, brain fogginess was reproduced in 66% of patients. This suggests that the carbohydrate meal provoked brain fogginess and reproduced their typical symptom experienced at home. Furthermore, 63% of patients had SIBO (as evidenced by duodenal aspirate/culture or breath test), and 80% had D-lactic acid/L-lactic acidosis. In contrast, SIBO and D-lactic acidosis was seen in a significantly lower proportion (28%) of patients with similar gastrointestinal symptoms, but without BF. These data provide compelling evidence for possible link between brain fogginess, SIBO and d-lactic acidosis. Unlike previous reports, our cohort developed d-lactic acidosis and BF without impaired renal or hepatic function and/or short bowel syndrome^3^.

D-lactic acidosis was first described by Oh et al in 1979 in a patient with short bowel syndrome who presented with unexplained anion gap metabolic acidosis and episodes of severe neuropsychological symptoms^2^. The L-isomer of lactic acid is the main form of lactic acid in the human body and is produced by the enzymatic (L-lactate dehydrogenase) reduction of pyruvate. D-lactate is produced in much smaller quantities by the enzyme D-2-hydroxyl acid dehydrogenase that also metabolizes D-lactate in the human liver^3^. Unlike L-lactate that is efficiently metabolized, the breakdown of D-lactate via the methylglyoxal pathway is slow and limited and can be confounded by comorbid conditions causing significant accumulation and acidosis^3,20^. In short bowel syndrome, if the colon is colonized by D-lactate producing bacteria, the delivery of large amounts of unabsorbed carbohydrate causes rapid fermentation, gaseous distension, and production of large amounts of d-lactic acid overwhelming hepatic clearance^20^, and causing D-lactic acidosis and encephalopathy.

Here, the BF was likely induced by the production of toxic metabolites such as D-lactic acid in the small intestine from bacterial fermentation of carbohydrate substrates. The use of prolonged or excessive probiotics and/or cultured yogurt further contributed to the small intestinal colonization by lactobacilli and other bacteria. These bacteria are generally resistant to usual antibiotics^3^, further explaining the refractory nature of symptoms.

Probiotics are considered to be safe and beneficial including improvement in gut barrier function and gut transit^21^. Although a meta-analysis of 57 studies indicated that probiotics are safe^22^, caution against its use has been recommended in subjects who are immunosuppressed, pregnant, and with structural heart lesions, acute abdomen, neutropenia, chemotherapy and radiotherapy^22,23^. However, studies have not been well designed, and safety outcomes have been inconsistently reported. The Agency for Healthcare Research and Quality concluded that there is inadequate literature on the safety of probiotics, especially given their widespread and OTC use^24^. There are reports of fungemia, bacteremia and gut ischemia in at risk, critically ill populations, many using *Saccharomyces* species or *Lactobacillus*^23–27^.

*Lactobacillus* species and bifidobacterium are the most common bacteria in probiotic formulations^21,28^, and are felt to be useful in the treatment of irritable bowel syndrome, inflammatory bowel disease, and other intestinal problems^29^. Both bacteria produce D-lactic acid. D-lactic acidosis has been described with *Salmonella enteritidis* and with probiotics^30^. Probiotics are designed to deliver bacteria to the colon but whether this is achieved has not been reliably shown^21–23,27,28,31^. In contrast, they may colonize the small bowel, especially in the presence of dysmotility or low acid conditions that favor bacterial overgrowth. We found a variety of aerobic and anaerobic species of bacteria in the proximal small bowel and three patients had Lactobacilli and/or Prevotella species. Also these bacteria are resistant to routine antibiotics. Interestingly, some *Lactobacillus* species such as *Lactobacillus* GG only produce L-lactate^30,32^. Hence, both D-lactate and L-lactate should be measured when assessing this condition, and the most practical way for its diagnosis is to administer a carbohydrate meal and assess D-lactate in urine and L-lactate in blood, over the next 3 h along with breath samples for hydrogen and methane.

We treated all patients with evidence of SIBO with antibiotics and discontinuation of probiotics, and the rest with dietary advice and stopping probiotics. These measures led to significant improvement of symptoms in 70% of our patients and complete resolution of brain fogginess in 85% of patients, reaffirming that the symptoms were related to D-lactic acidosis and SIBO. Likewise, the group of patients without BF, but with either SIBO or D-lactic acidosis also showed similar degree of improvement in symptoms after antibiotics.

One possible reason for bacterial colonization is intestinal dysmotility^10^ Similarly dumping or rapid upper gut transit may cause colonic fermentation similar to short bowel. We measured regional and whole gut transit time using validated techniques^14^. In the BF group, except one patient, none showed evidence for dumping. About 1/3rd of our subjects had gastrointestinal dysmotility including gastroparesis, delayed small bowel and colonic transit that may have contributed to SIBO^10,11,13,14^, but rest had normal transit. Likewise in the non-BF group, 25% had gastroparesis and 37% had slow transit constipation that may have contributed to SIBO, but there was no difference between the two groups suggesting that dysmotility by itself may not contribute to brain fogginess. Additionally, opioids and PPI use may have predisposed patients to SIBO as 78% of PPI users had D-lactic acidosis^10^.

Our study has several limitations including the observational nature, although we examined a consecutive series of patients. Also, we did not culture fluid from the distal small bowel where most of the colonization may have occurred from probiotics because of the practical limitation of reaching this portion of the gut using standard endoscopy. This may have limited our ability to accurately link the probiotic use with SIBO. We did not retest these subjects to check if SIBO had resolved. There is no medical definition or criteria for BF, and therefore we relied on patient’s description of symptoms, and used the presence of ≥2/5 symptoms suggestive of BF. Also, whether the BF group had SIBO prior to using probiotics is unclear.

In conclusion, we describe a syndrome of a constellation of symptoms that comprises of post-prandial abdominal bloating, distension, gas and BF that were associated with the use of probiotics, and possibly caused by SIBO and D-lactic acidosis. The prevalence of SIBO and D-lactic acidosis was significantly higher in patients with BF than those without BF. We advise caution against excessive and indiscriminate use of probiotics especially without a well-defined medical indication, and particularly in patients with gastrointestinal dysmotility, and/or those using long term PPI and opioids.

## Study Highlights

### What is current knowledge

- D-lactic acidosis is rarely encountered in routine GI practice, but occasionally seen in patients with short bowel syndrome.
- Recently, we have encountered several patients with unexplained brain fogginess, associated with significant gas, bloating and abdominal pain. Whether these symptoms are due to D-lactic acidosis and are associated with small intestinal bacterial overgrowth (SIBO) is not known.

### What is new here

- We found that over 2/3rd of patients with brain fogginess demonstrated D-lactic acidosis,along with significantly higher prevalence of SIBO when compared to those without brain fogginess.
- Discontinuation of probiotics along with the use of antibiotics led to resolution of symptoms. Clinicians should recognize, diagnose and treat this unique syndrome.
